# Supplementary figures and images for: Using strain-resolved analysis to identify contamination in metagenomics data
Source: Microbiome. 2023 Mar 2;11:36. doi: 10.1186/s40168-023-01477-2 (PMC9979413; doi:10.1186/s40168-023-01477-2)

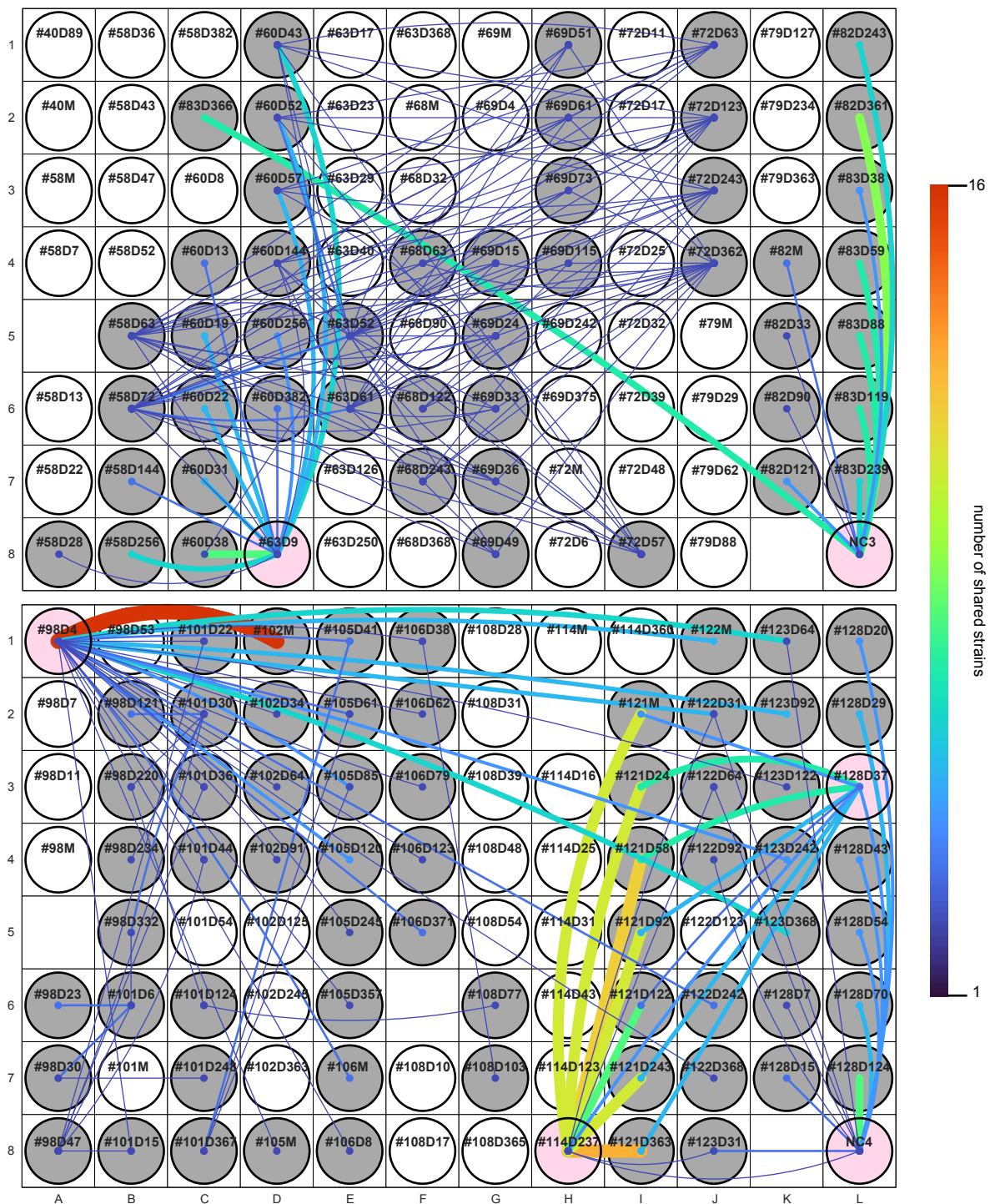

Supplement: Supplementary file 2 — Additional file 1: Figure S1. Details of strain sharing on P3 and P4 from case study I. Rectangular areas represent plates (P3 and P4) and circles show sample placements within each plate. Infant samples are named by infant number and infant day of life (i.e., #63D9 refers to infant #63 and this sample was collected when the infant was 9-day-old). If it is a maternal sample, such a sample is named by the infant number with a letter “M” in the end (i.e., #40M refers to the maternal fecal sample collected from infant #40). A line was drawn between unrelated samples if they shared ≥1 strain. The more strains a sample pair shared, the thicker and brighter the line. If a sample did not share any strains with other unrelated samples, its corresponding circle is colorless. Pink circles represent samples that were likely cross-contaminated. [file 40168_2023_1477_MOESM1_ESM.pdf]

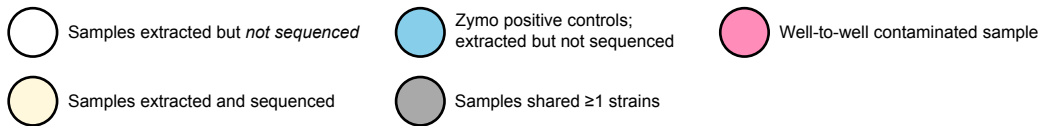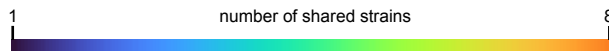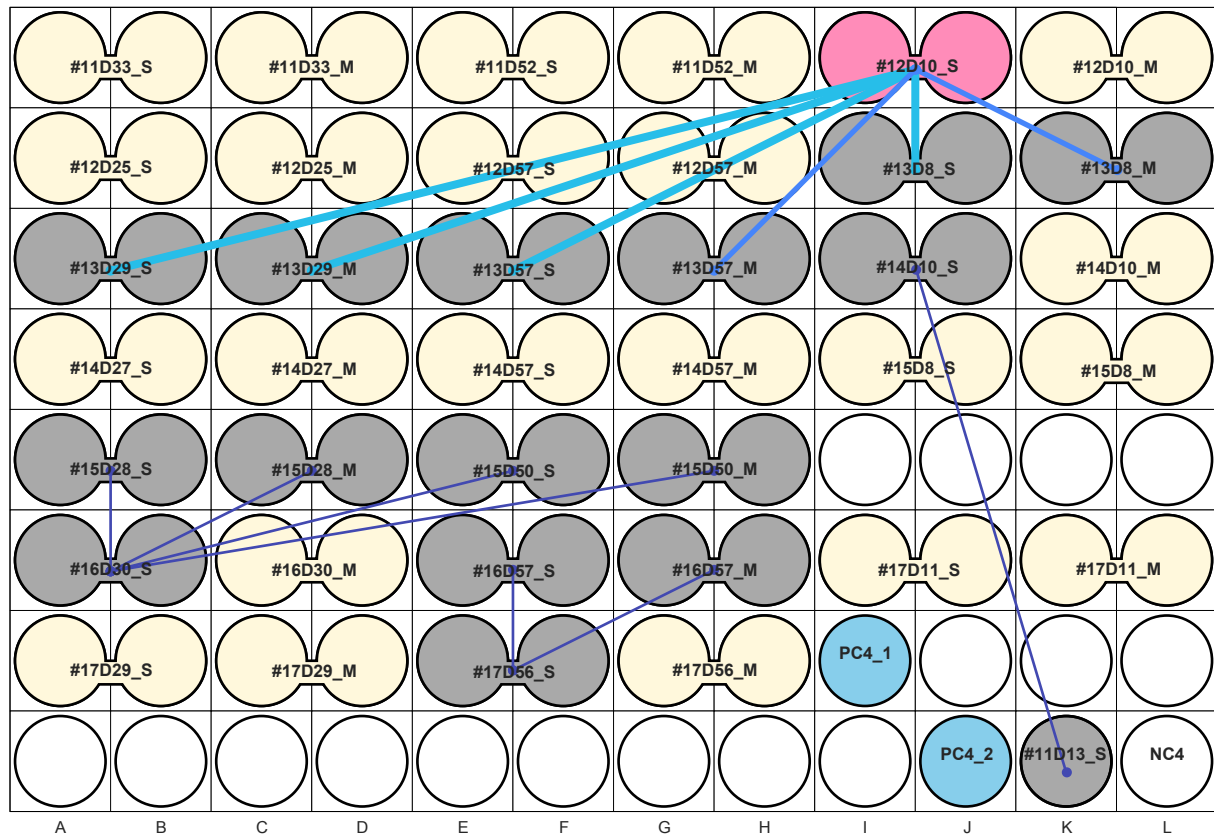

Supplement: Supplementary file 3 — Additional file 2: Figure S2. Detection of one cross-contaminated sample on P4 from case study II. Merged circles represent duplicated samples that were extracted adjacent to each other and were merged before being transferred to the library preparation plates. Infant samples are named by infant number, infant day of life and sample type (“M” refers to mouth samples, “S” refers to skin samples, and “G” refers to gut samples). If a sample pair from unrelated infants shared ≥1 strain, the corresponding samples circles were colored gray and a line was drawn between them. The more strains a sample pair shared, the thicker and brighter the line. [file 40168_2023_1477_MOESM2_ESM.pdf]
